# Searching for the genes that separate species

**DOI:** 10.7554/eLife.05377

**Published:** 2014-12-09

**Authors:** Megan Phifer-Rixey

**Affiliations:** 1 **Megan Phifer-Rixey** is in the Museum of Vertebrate Zoology and Department of Integrative Biology, University of California, Berkeley, Berkeley, United Statesmrixey@berkeley.edu

**Keywords:** speciation, reproductive isolation, hybrid zone, mouse

## Abstract

Hybrid mice shed new light on the interactions between regions of the genome that help drive the evolution of new species by reducing the fertility of hybrid males.

**Related research article** Turner LM, Harr B. 2014. Genome-wide mapping in a house mouse hybrid zone reveals hybrid sterility loci and Dobzhansky–Muller interactions. *eLife* **3**:e02504. doi: 10.7554/eLife.02504

**Image** Many regions all over the genome are associated with infertility in hybrid male house mice

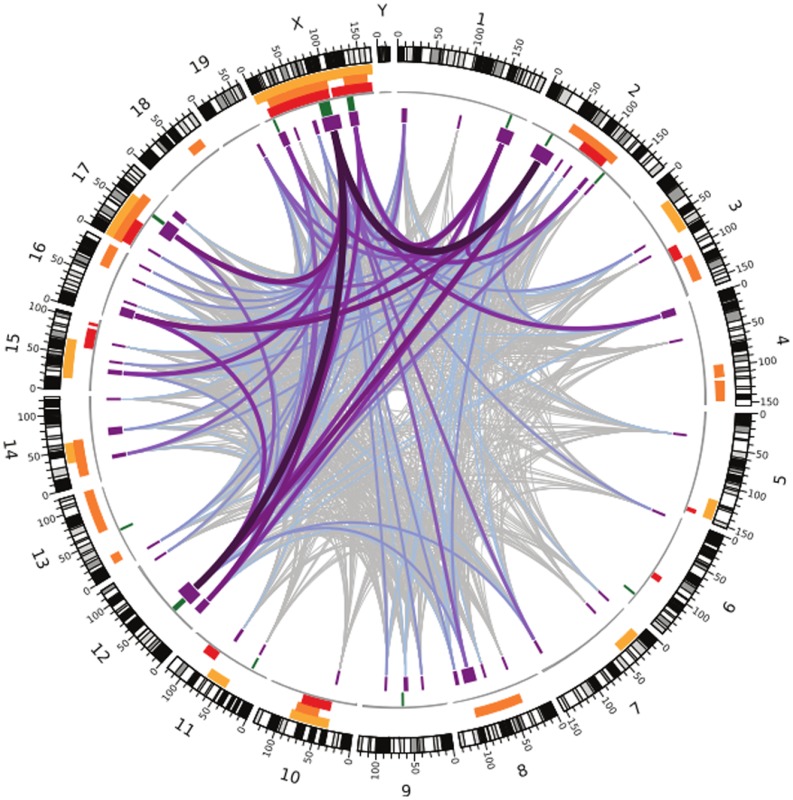

When members of the same species are separated into two populations that have no contact with each other, genetic differences accumulate over time. Later, if they come back into contact, the two populations may no longer be able to breed with each other or, if they can breed together, their offspring may be infertile. When this happens, the two populations are said to be reproductively isolated and they can be classed as separate species.

Of course, not all of the genetic differences between recently separated species contribute to reproductive isolation, and identifying the ones that do has been a major challenge. So far, relatively few genes that contribute to reproductive isolation have been found, and most of them come from the fruit fly *Drosophila* (Presgraves, 2010).

Now, in eLife, Leslie Turner and Bettina Harr of the Max Planck Institute of Evolutionary Biology report that they have developed a new approach to study reproductive isolation in house mice (Turner and Harr, 2014). The house mouse is well suited for these studies because there are three subspecies that separated relatively recently, around 350–500 thousand years ago (e.g., Boursot et al., 1993). In parts of Central Europe, two of the subspecies—*Mus musculus domesticus* and *Mus musculus musculus*—live alongside each other, and they can mate and produce hybrid offspring (e.g., Sage et al., 1986; Boursot et al., 1993). However, when the two subspecies are cross-bred in the laboratory, the hybrid males are often less fertile than the parents (e.g., Good et al., 2008; White et al., 2011).

Laboratory crosses have led to important insights into the evolution of new species. Like in other species, it is clear that the X chromosome plays a major role in causing hybrid male mice to be sterile (e.g., Good et al., 2008; Mihola et al., 2009; White et al., 2011). Moreover, reproductive isolation is not a simple trait that is caused by a few genes: it is due to the contributions of many genes throughout the genome (e.g., White et al., 2011). These findings agree with the results of studies of wild mice caught in the hybrid zone in which researchers examined the exchange of genetic variation between the two subspecies (e.g., Tucker et al., 1992; Payseur et al., 2004; Teeter et al., 2008; Janoušek et al., 2012).

While both of these approaches have been successful in finding regions of the genome that are responsible for reproductive isolation, identifying the specific genes involved, and how they interact with each other, remains a challenge. Over three decades of work using mapping and positional cloning techniques has only conclusively identified one gene that contributes to sterility in house mice, *PRDM9* (Mihola et al., 2009). The main problem is that the candidate regions identified using these approaches are large and include many genes, and it is painstaking work to test each of these individual genes. With such a long list of candidates, investing a high level of effort in any one gene is a gamble.

Now, Turner and Harr demonstrate a method that can narrow down the search for genes into smaller genomic regions. They carried out a genome wide association study on the offspring of wild mice caught in the hybrid zone. In the study, they looked for regions of the genome that were associated with variation in two indicators of male sterility: relative testis weight and gene expression in the testes. They also looked for interactions among the candidate regions they had identified.

Like the earlier studies, they found that many regions across the genome contribute to sterility in hybrid males, with strong evidence that regions on the X chromosome are involved. They analysed the data using several different methods, and by focusing on the regions that were highlighted by multiple methods, they were able to narrow down their list of candidate regions. Overall, they found nine regions were associated with variations in relative testis weight, and 50 regions that were associated with variations in testis gene expression (Figure 1).

**Figure 1.**
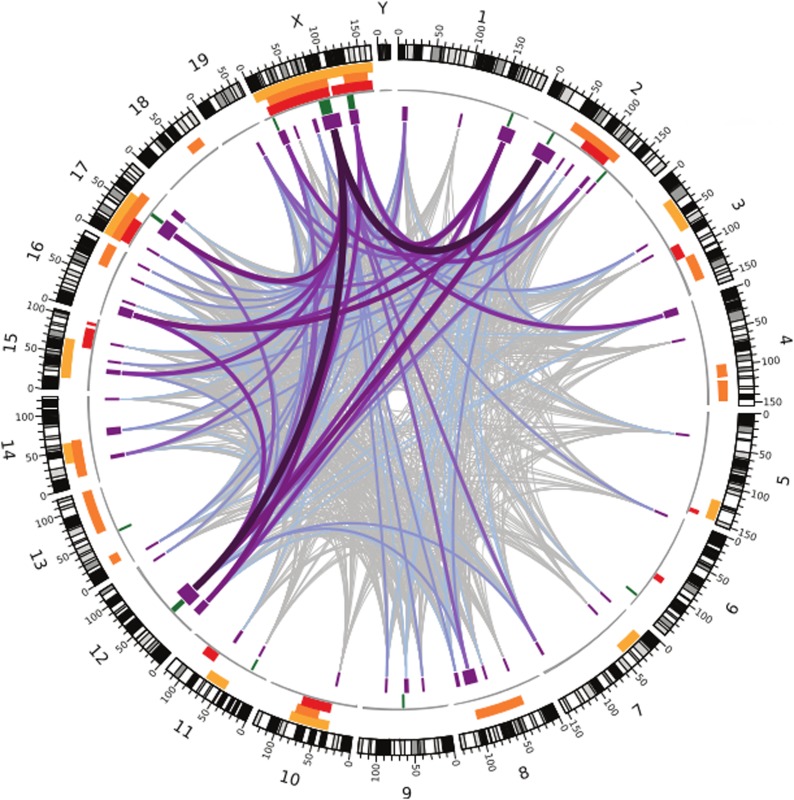
Many regions of the house mouse genome are associated with variation in the expression of genes in the testes, a trait related to male sterility. In this map—taken from Turner and Harr, 2014—the edge of the circle indicates the position in the genome along the chromosomes pairs 1–19 and the pair of sex chromosomes X and Y. The purple boxes indicate the regions that the genome wide association study found to be associated with gene expression in the testes of hybrid male mice. The lines show which regions interact with each other, and the color indicates how variable the DNA sequences of these regions are (grey represents high variability; deep purple represents high variability). The green lines indicate genome regions that were associated with variation in testis weight in the study. The orange and yellow boxes indicate the genome regions that have been previously identified using other approaches.

What makes this method powerful is the improved resolution, which makes it possible to identify smaller regions: the median size of candidate genome regions identified in this study is only 2 Mb, and many of these regions contain relatively few genes. This includes some genes that have no known connection to fertility, which might have been overlooked with a different approach.

This study suggests two ways forward. First, many of the candidate regions identified by Turner and Harr overlap with candidate regions found in previous studies. These regions would be promising starting points for future studies to identify the specific genes that contribute to hybrid male sterility in house mice. Second, the method could be used to study reproductive isolation in other organisms, where it would be difficult to use other approaches because we know less about their genomes. Understanding the genetics behind reproductive isolation in many species may reveal new insights into the evolution of new species that are currently hidden by the focus on a few well-known model organisms.
